# Deceit and facial expression in children: the enabling role of the “poker face” child and the dependent personality of the detector

**DOI:** 10.3389/fpsyg.2015.01089

**Published:** 2015-07-28

**Authors:** Marien Gadea, Marta Aliño, Raúl Espert, Alicia Salvador

**Affiliations:** Department of Psychobiology, Faculty of Psychology, University of València, ValènciaSpain

**Keywords:** deceit, children, facial expression, emotion, dependent personality, gender differences

## Abstract

This study presents the relation between the facial expression of a group of children when they told a lie and the accuracy in detecting the lie by a sample of adults. To evaluate the intensity and type of emotional content of the children’s faces, we applied an automated method capable of analyzing the facial information from the video recordings (FaceReader 5.0 software). The program classified videos as showing a neutral facial expression or an emotional one. There was a significant higher mean of hits for the emotional than for the neutral videos, and a significant negative correlation between the intensity of the neutral expression and the number of hits from the detectors. The lies expressed with emotional facial expression were more easily recognized by adults than the lies expressed with a “poker face”; thus, the less expressive the child the harder it was to guess. The accuracy of the lie detectors was then correlated with their subclinical traits of personality disorders, to find that participants scoring higher in the dependent personality were significantly better lie detectors. A non-significant tendency for women to discriminate better was also found, whereas men tended to be more suspicious than women when judging the children’s veracity. This study is the first to automatically decode the facial information of the lying child and relate these results with personality characteristics of the lie detectors in the context of deceptive behavior research. Implications for forensic psychology were suggested: to explore whether the induction of an emotion in a child during an interview could be useful to evaluate the testimony during legal trials.

## Introduction

The present study fits in the general background of the need to identify valid indicators of deceptive behavior and/or find measures that validly discriminate between liars and truth-tellers. For a long time, this question has stimulated interdisciplinary research despite criticism and skepticism due to the general lack of valid and reliable results as well as discussions around the utility of the laboratory-designs used to explore deception (Vrij and Granhag, 2012). One important claim in this field is to examine deception in quite naturalistic settings, with the aim to provide relatively unrestricted “honest vs. deceptive” statements and so improve ecological validity (Gamer and Ambach, 2014). A comprehensive definition of deception is that “succesful or unsuccessful deliberate attempt, without forewarning, to create in another person a belief which the communicator considers to be untrue” (Vrij, 2000, p. 15). Thus, the deceptive behavior is an interpersonal exchange of information: a very special one where there is a liar and a deceived person, or lie detector. In this kind of social communication, adult subjects achieve, overall, an accuracy rate of around 54% of all statements judged -independently whether they are truth or lie – only slightly above chance (see the meta-analysis of nearly 24,500 veracity judgements by Bond and DePaulo, 2006). The ability to differentiate between children’s true and false statements is also an important issue on this field because children can be victims or witnesses to crimes and may be required to testify about their experiences in court (Brunet et al., 2013; see also the review of Lee, 2013). The studies show that when adults attempt to differentiate children’s deceptive behavior, including parents, child protection lawyers, police and social workers, and judges, are highly inaccurate and rarely perform above chance levels (Crossman and Lewis, 2006; Eldestein et al., 2006). This fact has been partially explained by some authors by referring to the observed behavior of children when they lie, closely mimicked from subjects who are telling the truth (e.g., to make direct eye contact, Talwar and Lee, 2002).

Until present, no demographic individual difference (i.g., gender, education, age, experience) has shown to be reliably related to deception detection’s accuracy (Aamodt and Custer, 2006). It has been shown recently that people find limitations in lie detection mainly due to weakness in behavioral cues of deception (Hartwig and Bond, 2011). So the effort of researchers to improve lie detection should focus on increasing behavioral differences between the liars and the truth tellers. With some exceptions, few studies have focused on the liar to determine individual differences in the ability to lie and some authors have claimed the necessity to study the liar more in deep to fully understand the deceptive behavior (Wright et al., 2013), for instance, Wright et al. (2012) showed that the best lie detectors are also the best liars. Moreover, there are some physical characteristics and observed behaviors which have been associated to the liar, under the assumption that some hidden mental states associated to the act of lying could influence behavior and, therefore, the lie could be inferred (Hill and Craig, 2002). In this sense, certain parameters of the speech (Spence et al., 2012) or a number of kinetic variables (Duran et al., 2013) have been proven to be capable of differentiating between liars and truth tellers. One of the most studied parameters is the facial expression of the liar, given that the human face provides a number of signals which are essential for interpersonal communication in our social life. The face has been considered the place for the most expressive behavior and a window to the subject’s mental states, where people possibly cannot overcome the constraints of the translation of their intentions into their expressions (DePaulo, 1992). It has been recently shown through automated tools that when deceptive behavior spontaneously appears, continuous fluctuations of movement in the upper face are characterized by dynamical properties of less stability and greater complexity, despite no apparent differences in the overall amount of movement between deception and truth (Duran et al., 2013). The face is thought to be susceptible of a “leakage” of hidden negative emotional states (supposedly associated to deceptive behavior) that shows facial micro-expressions lasting only tenths of a second, which some authors claim to be a clue of deception (Ekman and Friesen, 2003). This could be especially clear when taking into account the type of lie, for instance, Warren et al. (2009) showed that the detectors were significantly above chance for emotional compared with unemotional lies ones, and they reported the benefit of subtle facial expressions of the liars as the key for the task. Thus, one can assume that the facial expression of the speaker when expressing emotions is a determinant factor for the possibility of detecting his/her deceptive behavior. Then one could wonder if every individual person perceives those emotional cues of the face equally, and if the answer is “no” (as is plausible) then maybe exists a detector whose perception is more adequate to detect lies. In fact, despite of the very poor average performance in lie detection, there are some persons that seem to be especially good at this task, as showed by Ekman et al. (1999), who found accuracy rates from 68 to 73% amongst groups with a special interest in deception detection (unfortunately, they did not measure the individual differences in emotional perception of the detectors). Of more interest is the finding of Ein-Dor and Perry (2014) regarding the attachment anxiety, but not other types of anxiety, predicted more accurate detection of deceitful statements. It is reasonable to think that subjects with attachment anxiety do not perceive the emotional information expressed by others in the same manner than the healthy persons. Moreover, we can expand the search a bit far from explicitly clinical disorders to subclinical traits. In the interesting work of Nettle (2006) regarding personality variations, the author argues the possible adaptive function of certain personality characteristics often viewed as undesirable, for instance, the benefits of neuroticism would be vigilance to dangers, striving and competitiveness (in front of its costs of stress, anxiety disorder, and depression). Other authors suggested that clinical levels of paranoia may represent the inevitable cost of efficient threat perception–or ‘justified’ suspicion – that is necessary for survival of the human species (Green and Phillips, 2004). So, the exploration of subclinical levels of these personality traits in the normal population in relation with lie detection could be interesting as well.

Given the above scenario, our main aim was to investigate the relation between the facial expressions of a group of children when they told a lie and the chance of detecting the lie by a sample of adult detectors. To evaluate the intensity and type of emotional content of the children’s faces, we applied an automated method capable of analyzing the facial information from the video recordings, the FaceReader 5.0 software. This is a tool whose neural network has been trained using a high-quality set of approximately 10,000 facial images, manually described as emotional or not by human coders, achieving a classification accuracy of 89% by itself (den Uyl and van Kuilenburg, 2005; Terzis et al., 2010). We expected that the information obtained from the FaceReader regarding the emotionality expressed in the children’s face would be positively related to the success in the lie detection task. A second aim was to test the possibility that the anxious or the paranoid personality disorder measured at a subclinical level in the sample of detectors could be related to a more accurate detection of interpersonal deceit.

## Materials and Methods

### Subjects

#### The Liars: Children Performing the Videotapes

A total of four Caucasian Spanish-speaking female students of 7 years-old were selected from a local school after obtaining informed consent from their parents. We asked for children in a classroom of a primary school which we had access through one of the teachers and with the permission of the headteacher. We got favorable feedback from the parents of only five children: the four girls plus one boy that we didn’t include in the sample in order to be homogeneous about the gender variable. This age was selected because the ability of lying, in the sense of applying an intentional component in the discourse to deceit, is supposedly reached. Theories of development regarding the ability of lying suggest it increases from 2 to 3 years to reach its pick at 6 years, in parallel to the development of Theory of Mind and executive functioning in children (Talwar and Lee, 2008; Evans and Lee, 2013; Cheung et al., 2015). The participants had no reported history of psychiatric disorders, medical illness or chronic pharmacological treatment, and their intellectual abilities were normative and homogenous according to their teacher.

#### The Detectors: Sample of Adults Who Watched the Videotapes

A total of 104 young adults, undergraduate volunteers between 18 and 26 years-old, 29 men with a mean age of 20.03 years (SD 4.07) and 75 women with a mean age of 19.91 years (SD 4.48), were selected from the University community to take part in the experiment, without monetary reward. Participants were excluded if they reported current or past psychiatric/neurologic illness, use of psychotropic medication or chronic pharmacological treatment.

All subjects were treated in accordance with “Ethical Principles of Psychologists and Code of Conduct”^1^. All procedures were in accordance with the standards of our institutional committee of ethics in research with humans that approves the experiments, and with the 1964 Helsinki declaration and its later amendments.

### Materials

#### Recording of the Videos: the Deceit Detection Test

The Deceit Detection Test consisted in 12 video-recordings that expressed true or false statements from the group of female children cited above. This material was elaborated as follows.

The videos were filmed with a Sony Handycam HDR-PJ200E in a well illuminated room of our laboratory. Special care was taken to ensure good frontal light on the participant’s face, which is an important requirement for the FaceReader 5.0 software to produce reliable results. Also important is that participants are looking directly toward the camera while showing their facial expression. Although the software can handle rotations up to 40°, minimal rotation is desired to ensure optimal quality readings. The recordings with a resolution of 320 × 240 at 12 frames per second were saved as AVI files to be analyzed later with FaceReader 5.0 software. The videotapes were conducted by two researchers who were unaware of the aim of the experiment. Once the context of the recording was set, one of the researchers instructed the children about the task. The children were told they must tell a story, freely chosen by them. The story must be about what happened at a particular moment in time, and must be false or true. So, they were asked to tell the truth or elaborate a spontaneous lie about what happened in a concrete temporal moment of their life (past, present, future). Meanwhile, the other researcher took the record with the camera (six videos for each child, being three with false content and three with true). The children were pressed a bit to do so (and to do it right). This was achieved when one researcher (a stranger man for them) with a camera in hand pointing to her face told them that the acting was “very important for their parents and they must perform the show correctly.” The reader can consult concretely the given instructions and a transcription to English of the valid recordings in the Supplementary Material section. It has been argued that to give fixed instructions about when to lie (and when not to) means that the experimental study lacks of ecological validity (Sweeney and Ceci, 2014), but our aim was that the situation resembled a demand to the children to follow instructions from the authority (obey to the parent in the occurrence of a legal trial for instance) given the data pointing that children are very able to fabricate false reports to gain some advantage or to satisfy authority (Bala et al., 2005). These procedures resulted in 24 naturalistic and spontaneously performed videos with a maximum duration of about 1 min each. A pre-selection of the videos was carried out before implemented the experiment: note that the children were free to tell what they want, and some of their statements were inadequate (the instruction was to give a plausible lie, so statements like “I go to the moon this afternoon” were considered invalid) and other were too short or not easily understood. Thus, an individual and separated evaluation of them was additionally performed by three clinical judges with the aim of selecting the best videos in terms of general credibility, veracity and realism, speech quality and similar duration (length). This resulted in 14 videos (seven genuine, seven deceptive) that lasted an average of 32 s with a minimum of 12 s and a maximum of 64 s (SD: 15.7). The minimum number of videos for a given child was two and the maximum, five. These videos were submitted to the FaceReader analysis but unfortunately two of them could not be evaluated by the software due to technical reasons, so the final data analysis was performed on 12 videos (six truths and six lies, minimum number of videos from the same child one, maximum four) that were included in the test. These videos were presented in a computer to the sample of detectors, sequentially and in semi counterbalanced order. After each video a gray screen with genuine or deceptive options was shown (“was the girl telling a lie or telling a truth?”) so the detector could complete a direct, self-report judgment. The scoring of the videos is explained in next sections.

#### Automated Analysis of Facial Expression: FaceReader 5.0

Facial expressions were analyzed using FaceReader software version 5.0 (Noldus Information Technology B. V., 2012), a commercially available program that uses algorithms to evaluate and classify, on frame-by-frame basis, facial images and videos into the following categories of basic emotions: happiness, sadness, anger, surprise, fear, disgust, and neutral (Ekman, 1970). FaceReader works in three steps: (1) face finding, (2) face modeling, and (3) face classification. This tool finds a face using the Active Template Method. Then it creates a virtual, super-imposed 3D Active Appearance Model featuring almost 500 unique marks of the face. In a third stage, scores for the intensity and probability of facial expressions for basic emotions are computed. These variables reflect a measure of the magnitude of that emotion being shown from 0% (not at all) to 100% (perfect match). In our study, this facial analysis software analyzed more than 370 s of video recording, i.e., around 5,685 frames on six basic emotion scales. In FaceReader you can choose from a list of four models to fit (general, children, east-asian, and elderly) so the appropriate model was selected (Caucasian children between 3 and 10 years). Additionally, a variable that FaceReader takes into account consists in the characteristic facial expression that some people have by nature (sad, angry, etc.). You can calibrate FaceReader to correct for these person’s specific biases toward a certain facial expression so that a real emotion can be analyzed. To do so, the user must use one or more videos as calibration material, as it was done in the current study. In the current research, at least two videos of each person were chosen for the calibration process, and a higher sample rate was implemented, because our video’s length did not exceed the minute. This was to make sure that the calibration material contained a diverse set of images.

After the analysis we classified each video in just two categories: “neutral” or “emotional.” The “neutral” videos were those whose percentage without emotional expression (neutral) was between 70 and 94%. The emotional videos were when the sum of all the expressed emotions was higher than the percent of neutral expression (thus note that emotional videos could include some more “happy” and other more “sad”). This classification resulted in six emotional videos and six neutral videos, being half of them a lie and half of them a truth, respectively.

#### Personality Disorders Screening Test: Salamanca Questionnaire

Recently published by Pérez Urdániz et al. (2011) as a screening tool to evaluate 11 personality disorders, some of them according to The Diagnostic and Statistical Manual of Mental Disorders (DSM) version IV-TR (paranoid, schizoid, schizotypal, histrionic, antisocial, narcissist, and dependent) and some other according to the International Classification of Diseases (ICD) version 10 (emotionally unstable personality disorder-impulsive type, emotionally unstable personality disorder-borderline type, also known as limit, anankastic, and anxious.). Additionally, the 11 traits are categorized in three different groups: Type A: strange and extravagants (paranoid, schizoid, and schizotype), Type B: immature (histrionic, antisocial, narcissist, and both subtypes of emotional unstable disorders: impulsive and limit), and Type C: avoiding (anankastic, dependent, and anxious). The questionnaire consists in a total of 22 questions and each trait of personality is evaluated trough two questions with a 4-point Likert scale (false = 0 points; sometimes true = 1 point; usually true = 2 points; always true = 3 points). The cutoff score is established at three points for every trait. This questionnaire has been validated and correlated with the Interpersonal Personality Disorder Examination, being considered an adequate test of screening, with a sensitivity of 100% and a specificity of 76.3% (Caldero-Alonso, 2009). It is a self-assessment questionnaire (<10 min) with an easy interpretation.

### Dependent Variables and Statistical Analysis

Regarding the Deceit Detection Test, seven raw dependent variables were considered for the analyses: (1). Total Hits, is the total score when the detectors guess the child’s statement (both the true and false) with a maximum of 12. (2). False Positives, when the detectors thought that a statement was genuine but it was deceptive (the detector believed in the girl but she was lying) with a maximum of six. (3). False Negatives, the total score when detectors thought the statement was deceptive but it was not (the detector did not believe in the girl but she was telling the truth) with a maximum of six. (4). Deception-Hits: we considered separately the scores of the detectors regarding the false statements, with a maximum of six, and the (5) Truth-Hits: the scores of the detectors regarding the true statements, with a maximum of six. It was also considered what was guessed according to the FaceReader analysis: (6) Emotional-Hits (with a maximum of six) for the guessed about videos with emotional content; and (7) Neutral-Hits (with a maximum of six) regarding the videos without emotional content.

We used also signal-detection analysis for hypothesis testing (Stanislaw and Todorov, 1999), by calculating the discriminability (d’) index and the participantbias criterion (C) index, regarding their ability to detect the lies. The interpretation of (d’) is that the larger the index the better the discriminability, where values near 0 indicate random performance. When the (C) index is 0, this indicates no bias in the judge. Being the signal a lie, a negative (C) index indicates a truth-bias and a positive one indicates a lie-bias. On the other hand, The Salamanca Questionnaire gave 14 scores: Three main scores (for the main Type A, B, and C scales) and 11 subscores for each of the personality disorders described above.

All raw scores were mostly analyzed with non-parametric statistics due to the nature of the variables (Kolmogorov–Smirnov Test <0.05 in most cases). Thus, differences between related variables were tested with the Wilcoxon Sign-Rank Test, gender differences were tested with the *U* Mann–Whitney test, and associations between variables were tested with a series of Spearman Rank correlations. All analyses were run with SPSS 19. Data are presented in means, SD, confidence intervals, and index *d* Cohen for effect size when corresponding.

## Results

The **Table 1** shows a descriptive of the 12 videos recorded, with percent of each emotion, total sum of emotions and neutral expressions according the FaceReader analysis, as well as the classification of each video in Neutral or Emotional (N vs. E) and in True or False content (T vs. F), and the Total Hits (raw score and percent) observed for each video. There was a significant negative correlation between the intensity of the neutral expression (% Neutral/FaceReader) and number of hits from the sample (ρ = -0.70; *p* < 0.01). The videos were then classified in a 2 × 2 Table, according the T/F and E/N variables, to form four boxes (three videos in each). The mean percent of Hits for the three Emotional-True videos was 77.2%, and for the Emotional-False videos was 84.9% The mean percent of Hits for the three Neutral-True videos was 56.7% and for the Neutral-False was 46.4%. Thus, the percent-difference of correct classification for the true videos depending on emotional expression was 20.5%. For the false videos -depending on emotional expression as well- was 38.4%. This difference was tested with a Chi-square test but it was not significant [χ^2^ (gl 1) = 1.42; n.s.].

**Table 1:** General characterization of the videos: “Video/Girl” refers to number of the video and first initial of the name of each girl who was recorded.

VIDEO/GIRL	Happy	Sad	Surprised	Fear	Other	% Emo	% Neu	E/N	T/F	Total hits	% Hits
1 (A)	67.5%	6.3%	–	–	1.2%	75%	25%	E	F	85	81.7
2 (S)	53.5%	–	9%	–	0.3%	62.5%	37.2%	E	F	88	84.6
3 (A)	20.4%	–	–	–	1.4%	21.8%	78.2%	N	F	14	13.4
4 (F)	–	–	29%	–	–	29%	70%	N	V	54	51.9
5 (F)	–	–	20.9%	–	1.7%	22.6%	77.4%	N	F	83	79.8
6 (S)	–	–	5.9%	–	1.6%	7.5%	92.5%	N	V	75	72.1
7 (S)	–	–	55.2%	–	1.6%	56.8%	43.2%	E	F	92	88.4
8 (F)	–	–	53.9%	–	4.1%	58%	42%	E	V	86	82.6
9 (S)	6.1	–	22%	–	0.7%	28.8%	71.2%	N	F	48	46.1
10 (F)	47.1%	10.6%	15.7%	7.9%	0.2%	81.5%	18.5%	E	V	84	80.7
11 (L)	43.5%	–	–	8.2%	4.3%	55.9%	44%	E	V	71	68.2
12 (A)	–	–	–	–	5.8	5.8%	94.2%	N	V	48	46.1

*“Happy…Other” refer to observed percent of that emotion in the video from the FaceReader analysis.” % Emo” and “% Neu” refer to the observed total percent of expressed emotions and neutral expression from the FaceReader analysis. “E/N” refers to the classification of the videos into Emotional or Neutral. “T/F” refers to the classification of the videos into True or False content. Total Hits refers to the number of subjects (from the total *n* = 104) who guessed the content of each particular video, transformed into percent in the last column % Hits.*

The **Tables 2** and **3** show the means and SD for the sample (as a whole and separated into women and men) regarding the Deceit Detection Test, including the indexes of discriminability (*d*′) and the participant bias criterion (C), as well as the scores from the Salamanca Questionnaire as explained above (3 main scales and 11 subscales). The mean of Total Hits for the whole sample of videos (regardless of the content of the video) was 7.98 (SD: 1.4), so the classification was correct in a 66.5% of the recordings (which was significantly different from a constant of 50% chance: *t*(gl 103) = 14, *p* < 0.001). The difference between scores for False Positives (mean = 2.05; SD = 0.9) and False Negatives (mean = 1.96; SD = 1) was not significant, as neither was the difference between scores for Truth Hits (mean = 4.02; SD = 1.0) and Deception Hits (mean = 3.96; SD = 0.9). Interestingly, the difference between scores for Emotional Hits (mean = 4.88; SD = 0.9) and Neutral Hits (mean = 3.11; SD = 1.1) was significant (Wilcoxon *Z* = -7.7; *p* < 0.001; CI 95% for the mean Emotional-Hits (4.69–5.05) versus Neutral-Hits (2.88–3.32), d Cohen = 1.7).

**Table 2:** Means (Standard Differences) are shown for the Total sample (T) and separately for men (M) and women (W) for the scores of the Deceit Detection Test, including statistical trends for the gender variable.

	TH	FP	FN	TH	DH	EH	NH	*d*′	C
T (104)	7.98 (1.4)	2.05 (0.9)	1.96 (1)	4.02 (1)	3.96 (0.9)	4.88 (0.9)	3.11 (1.1)	0.33 (.24)	0.16 (.28)
M (29)	7.72 (1.4)	2 (0.9)	2.28 (0.9)	3.72 (0.9)	4 (0.9)	4.93 (0.9)	2.79 (1)	0.28 (.23)	0.23 (.26)
W (75)	8.08 (1.4)	2.07 (0.8)	1.84 (1)	4.13 (1)	3.95 (0.8)	4.85 (0.9)	3.23 (1)	0.35 (0.24)	0.13 (0.28)
*U*-Mann–Whitney	n.s.	n.s.	0.7	0.08	n.s.	n.s.	0.08	n.s.	n.s.

*TH, Total Hits, independent if the video performed a truth or a lie. FP, false positives (subject believed but the girl was lying), FN, false negatives (subject didn’t believe but the girl was telling the truth), TH, Truth Hits score: hits for true statements, HD, Deception Hits score: hits for false statements, EH, Emotional Hits: hits for videos with high emotional facial expression, HN, Neutral Hits: hits for videos with neutral facial expression, d’, index of discriminability, C, participant bias criterion index.*

**Table 3:** Means (Standard Differences) are shown for the Total sample (T) and separately for men (M) and women (W) for the scores of the Salamanca Personality Disorders Test, including statistical differences for the gender variable.

	SA	SB	SC	P	SD	ST	HS	AS	N	I	L	AN	D	ANX
T (104)	3.38 (2)	7.61 (2.9)	6.45 (2.6)	1.31 (0.95)	1.51 (1.3)	0.64 (1)	2.72 (1.3)	0.30 (0.6)	0.81 (0.9)	2.25 (1.3)	1.55 (1.2)	1.59 (1.3)	2.30 (1.4)	2.60 (1.4)
M (29)	4.52 (1.8)	8.07 (3.1)	5.97 (2.5)	1.66 (0.9)	1.97 (1.3)	0.90 (0.9)	2.66 (1.5)	0.45 (0.6)	1.17 (1.1)	1.86 (1.3)	1.93 (1.3)	1.59 (1.1)	1.86 (1.5)	2.52 (1.3)
W (75)	2.93 (1.9)	7.43 (2.8)	6.64 (2.6)	1.17 (0.9)	1.33 (1.3)	0.55 (1)	2.75 (1.2)	0.24 (0.5)	0.67 (0.8)	2.40 (1.3)	1.40 (1.1)	1.59 (1.4)	2.47 (1.3)	2.63 (1.4)
*U*-Mann–Whitney	0.001	n.s.	n.s.	0.01	0.02	0.008	n.s.	0.03	0.03	n.s.	n.s.	n.s.	0.04	n.s.

*SA-B-C, Salamanca Test; Type A-B-C scales, Subscales from the Salamanca: P, paranoid; SD, schizoid; ST, schizotype; HS, histrionic; AS, antisocial; N, narcissist; I, impulsive; L, limit; AN, anankastic; D, dependent; ANX, anxious.*

The pattern of significant correlations between the Deceit Detection Test and the Salamanca questionnaire showed a significant correlation between the Type C scale and the Neutral Hits score (ρ = 0.21, *p* < 0.02). The Dependent subscale (part of the Type C scale) was the most strongly related to the Deceit Detection Test, with significant direct correlations with the Hits score (Rho = 0.25, *p* < 0.008), with the Deception Hits score (ρ = 0.26, *p* < 0.007), and with the Neutral Hits score (ρ = 0.27, *p* < 0.003), as well as a negative correlation with the False positives score (ρ = -0.25, *p* < 0.009).

Despite the low number of men in the sample, we checked gender differences (see **Tables 2** and **3**) and observed a number of tendencies that approached significance in the Deceit Detection Test, especially the higher mean for Truth Hits in women (Mann–Whitney *U* = 858, *p* = 0.08) and the higher mean for Neutral Hits in women (Mann–Whitney *U* = 859, *p* = 0.08), as well as the higher mean for False Negatives in men (Mann–Whitney *U* = 853, *p* = 0.07). The differences for the Salamanca Questionnaire were more salient: men showed a significantly higher score in the Type A scale [strange and extravagant; Mann–Whitney *U* = 605, *p* < 0.001; CI 95% mean for men (3.8–5.2) vs. women (2.4–3.3), *d* Cohen = 0.8] as well as in each of its subscales: paranoid (Mann–Whitney *U* = 770, *p* < 0.01; CI 95% mean for men (1.3–2) versus women (0.9–1.3), d Cohen = 0.46), schizoid (Mann–Whitney *U* = 795, *p* < 0.02: CI 95% mean for men (1.4–2.4) versus women (1–1.6), d Cohen = 0.49), and schizotype [Mann–Whitney *U* = 769, *p* < 0.008; CI 95% mean for men (0.5–1.2) vs. women (0.3–0.7), *d* Cohen = 0.35]. Men also showed a significantly higher score in two subscales from the Type B scale (immature): antisocial [Mann–Whitney *U* = 873, *p* < 0.03; CI 95% mean for men (0.2–0.6) vs. women (0.1–0.3), *d* Cohen = 0.34] and narcissist [Mann–Whitney *U* = 815, *p* < 0.03; CI 95% mean for men (0.7–1.6) vs. women (0.4–0.8), *d* Cohen = 0.48]. On the other hand, women showed a significantly higher score in the dependent subscale of the Type C [Mann–Whitney *U* = 816, *p* < 0.04; CI 95% mean for men (1.2–2.4) vs. women (2.1–2.7), *d* Cohen = 0.42].

## Discussion

In the light on the outcome of the present experiment, our detectors were quite successful in determining the children’s truths and lies, since the classification was correct in 66.5%, significantly above from the standard 50–54% accuracy level (Bond and DePaulo, 2006), without significant differences between the detection of true or false videos, and with a moderately good overall index of discriminability. Studies that apply a paradigm in which the children choose to lie (or not) about a transgression by telling “no” (or “yes”) to the question: “did you peek?” show that adults are bad detectors, with both deception and truth detection near chance (Leach et al., 2004; Crossman and Lewis, 2006). Interestingly, some contextual variations of the task, like pressing the children to consider the moral implications of deceit or to promise to be honest before the task can facilitate the subsequent deception detection above chance (Leach et al., 2004). Our children were pressed to perform the task well to satisfy their parents, but they told us far more than a monosyllable: in our paradigm they must invent spontaneous stories (see transcriptions, Supplementary Material). Then, one could consider the speech’s content as a clue factor to perform the detection task. However, as explained before, care was taken in the selection of the videos to keep the children’s discourse reasonable even when they told a lie, so we should assume other factor out of the purely verbal contents of the discourse. Methods of veracity detection that use the linguistic differences in true and false stories (CBCA, reality monitoring) show rates of correct classification from 65 to 90% in trained detectors, but these methods when applied to children are problematic because children’s reports tend to contain fewer details and are generally shorter (Brunet et al., 2013). Most of the spontaneous reports of our children were in fact too short to apply CBCA. Another factor to consider could be the age of the children: were them too young to elaborate a good fake, so they were easily detected? We don’t think so, because from the point of view of the development of the ability of lying, a child of 7 years has reached enough level of Theory of Mind to be able to perform successfully lying, with intentionality and conventionality (Talwar and Lee, 2002; Lee, 2013).

Instead of the children’s age or the verbal content of their discourse, our interest was their facial expression as analyzed with an automated method: the FaceReader. The results were quite interesting: some videos were more easily guessed than others, whether the girl had told a truth or a lie (see **Table 1**). A significant inverse correlation between the accuracy in the detection and the neutral expression of the children appeared. Thus, the less expressive was the child the harder was the detection. This was confirmed when testing accuracy between the emotional and neutral videos: the mean success rate for the emotional videos was significantly higher (see **Table 2**). In addition, though non-significant, there was a 39% difference in guessing between the lies expressed with and without emotional facial expression (the “poker face” was harder to read). This makes the present methodology promising for future studies with higher samples. Some authors have analyzed differences between facial expressiveness of liars and control children before. For instance, in the study of Talwar et al. (2007) that variable was coded manually (non-automated) according the Facial Action Coding System (FACS, Ekman et al., 2002) and revealed small but significant differences between liars and control children in terms of both positive and negative facial expressions (unfortunately the authors did not tested its influence in the detectors). A more similar decoding than ours, with a computer-based automatic vision system, did recognize, with 85% accuracy, the facial expressions of faked pain in adults, compared to the recognition of trained human detectors, who obtained just 55% accuracy (Bartlett et al., 2014). Note that this data prove the existence of certain facial expression associated to deception that can be identified through automatic tools. In the present experiment the most emotionally expressive faces as automatically coded were the most transparent to human detectors, which helped for the detection of either false or true stories of the children. Some authors have suggested that emotional expressiveness in general is related to being judged as trustworthy (Boone and Buck, 2003); instead, we found it to be related as being more easily understood (including the hidden intentions to deceit). Following authors who suggest that the lies can be more accurately detected when less-conscious mental processes are used (Reinhard et al., 2013; ten Brinke et al., 2014) it is plausible that such unconscious process involving the perception of emotion in the face could facilitate the detection of deceptive or truthful information; this hypothesis remains to be evaluated. In addition, we observed a bias toward lies in our detectors: a positive C index indicating the labeling of the truth-tellers as liars. This supports the data of Crossman and Lewis (2006) about a suspicion in the detectors when evaluating children, judging them more prone to lie, which differs from research on detection of adult’s lies, that tends to demonstrate a truth bias (Eldestein et al., 2006).

An interesting finding of the present experiment was the relation between personality variables and lie detection. Among other personality traits studied, the attachment anxiety, described as anxiety from separation and abandonment, has been related to good lie detection as commented in the introduction. The attachment anxiety is related to the activation of a psychobiological innate system that motivates people to seek proximity to significant others if in need of protection from threats, and is known to be related to superior abilities to quickly and accurately detect of those threats and dangers (Mikulincer and Shaver, 2007; Ein-Dor et al., 2010). This raises the possibility for the existence of an innate ability to detect deceit, as a socially oriented threat, in these patients. Ein-Dor and Perry (2014) demonstrated that attachment anxiety (but not other types like social, avoidance, or security anxiety; DePaulo and Tang, 1994) predicted a more accurate detection of deceitful statements and a greater amount of money won during a poker game. Here we found that those subjects scoring higher in the dependent personality scale were significantly the most accurate in the task of detection and intriguingly also in the detection without facial emotional cues (the “hard” situation), as well as were less prone to believe that the statement was true when the girl was telling a lie. The definition (according DSM-IV-R) of the dependent subject as “showing passivity so that others take responsibility over the subject’s own decisions, along with subordination and inability to fend alone due to lack of confidence” is very close to that of attachment anxiety and, in fact, most patients with a dependent personality disorder have suffered from attachment anxiety in their childhood (Silove et al., 2011). The present results, along with data from Ein-Dor and Perry (2014), show that the attachment anxiety and the dependent personality at a subclinical level could offer certain social adaptive advantages. This supports the view of Nettle (2006) about variations in personality, which are better described in terms of a mixture of costs and benefits for the individual such that the optimal value for fitness may depend on a concrete context. In fact, literature shows that individuals with dependent personality disorder are very efficient at reading subtle social cues such as facial expression, presumably due to their need to behave in a way that maximizes probability of care (Bornstein, 2012); the present data are in complete accordance with this view. None other scale was related to lie detection, so we cannot support the view of Green and Phillips (2004) about adaptive advantages of paranoia, at least at a subclinical level and in relation with children’s lie detection.

Additionally, despite our sample had a low number of men, we were interested in testing gender differences. There is a general assumption that women are superior to men in interpreting other people’s non-verbal behavior (Hall, 1978). Women have advantage over men in reading facial expressions (DePaulo et al., 1993) though literature indicates that they have just an advantage when the person whose lies are trying to detect is a closer person, for instance a romantic partner (Vrij, 2011). No statistical differences were found here, only a trend for women to a better discrimination when judging a true statement and, interestingly, in the more difficult condition (neutral expression). This finding resembles the data of Wojciechowski et al. (2014) about the superiority of women in the performance of a deception task with inconsistencies between the facial and verbal cues. It would be of worth for future studies to perform more experiments with larger samples to check if women can be better lie detectors than men in a variety of harder circumstances (for instance, in total absence of facial emotional cues). In addition, we observed that men tended to be more suspicious than women when judging the children’s veracity, and their lie bias was higher, supporting the suggestion of DePaulo et al. (1993) about women being more inclined to believe that they are being told the truth than men. The reader must note that these latter assumptions are based only in statistical trends and are commented only to encourage other authors to check for gender differences routinely. Additionally, personality differences by gender showed men scoring significantly higher in paranoid, schizoid, schizotype, antisocial, and narcissist, and women scoring significantly higher in dependent, results that are in accordance with published data (Golomb et al., 1995; Bornstein, 1996).

In sum, we found that children telling deceptive or truthful stories with an unemotional facial expression according the FaceReader were harder to catch. Thus, the automated analysis of facial expression can help as a tool for detecting deception in children. In addition, the emotional expressiveness could affect stronger in some people with especial personality traits who possibly process the emotional info in a different way, concretely persons with subclinical dependent personality disorder; making them best lie detectors. This study has a number of limitations that could be overcome in future work. The number of videos applied was low and should be increased at least until a valid sample of videos of each of the basic emotions would be reached. The sample of detectors should be increased and be gender-matched. In addition, the detectors should be questioned about their perception of emotion in the facial expression of the children, how difficult they found the task, if they used the emotional or other clues to the task etc. At the moment from the present data, implications for forensic psychology are suggested: to explore whether the induction of an emotion in a child during an interview could be useful to evaluate the testimony during legal trials. In any case, these results and its implications are relevant specifically for those legal situations in which an adult pushes a child to tell a lie, but not necessary for those in which the child spontaneously decides to tell a lie. In addition, it should be noted that the children who go through a real legal process have much more motivation and their emotional state is much more complex, which could potentially affect adults’ ability to detect lies. In any case, further study of facial emotional expressiveness of children is of interest to forensic psychology.

## Conflict of Interest Statement

The authors declare that the research was conducted in the absence of any commercial or financial relationships that could be construed as a potential conflict of interest.

## Footnotes

1 http://www.apa.org/ethics/code/principles.pdf
