# Supplementary material for: Deceit and facial expression in children: the enabling role of the “poker face” child and the dependent personality of the detector
Source: Front Psychol. 2015 Jul 28;6:1089. doi: 10.3389/fpsyg.2015.01089 (PMC4516807; doi:10.3389/fpsyg.2015.01089)
Supplement: Supplementary file 1 [file Presentation_1.PDF]

Transcription to English of the verbal content of the videos (plus some more info)

There were two types of instructions. At first, the participants were asked to tell something real about what have they done in each of the following moments: a story for the past (the previous weekend), a story for the present: what they were doing in the present (the experiment was conducted on Saturday, so the child was allowed to tell things about that day) and a story for the future: what they were going to do after the experiment (the next Sunday, Monday or far more). In a different round, we asked her to tell a false story with the same temporal scope (past, present, future). We told them that their parents were waiting for them outside the lab and that the task was very important for their parents so they must accomplish the instructions correctly and perform a good show.

The girls were free to tell what they want and they did not have previous practice with the task, so the videos turned out to be very spontaneous, which we think it was very good to improve the ecological validity of the experiment.

Video number 1. (duration 1:02'')

"Hello, my name is XXX... I am going to tell something... I went for a fast food and they gave me... I asked for... a bit of apple with strawberry and banana... they gave me... they told me...that it just lasted in a minute, and they gave me a toffee as a dessert, and I told I didn't want that, and I was angry and........ later in my home, I didn't have that meal so I went again to the fast food but they gave the meal to a woman, and the woman wanted the toffee, then we changed our dishes and that was all".

More info: the story was false, totally invented; the girl paused a lot and stuttered a bit, though the discourse was comprehensible.

Video number 2 (duration: 0:27")

"Hello my name is XXX and tomorrow I'm going to Japan. I think they are going to tell me a story, and this is the story that some problem is coming so I have to go.

-Do you want to tell us anything else?

No..."

More info: the story was false. The discourse was comprehensible but the girl stopped talking so the experimenter asked her that question.

<u>Video number 3</u> (duration: 0:33")

"Last Saturday and Monday at the playground I played rope-jumping and after this I played with my skateboard which I like a lot because it seems like a bike because it has a bike brake.... I also can spin around and I have another skate with light in the wheels... Santa gave it to me."

More info: we suspected that the detail of the rope-jumping playing was true, but the girl didn't have skateboard, so the video was approved as false.

<u>Video number</u> 4 (duration: 0:13")

"On Sunday I went to the church and then I went to my home and I did my homework"

More info: this was a true story though unfortunately the girl went on no more and the experimenter did not react. We decided to go ahead with this video despite its short duration.

<u>Video number 5</u> (duration: 0:32")

"Tomorrow I'm going to the playground to play hide-and-seek

- *Are you going with someone?*

Yes, with XXX. We will play basket too. In the afternoon I'm going to the amusement park with XXX, XXX, and XXX."

More info: Again, this girl stopped talking, so she was asked for more content. All what she told was false according to the plans of her mother.

<u>Video number 6</u> (duration: 0:30")

"To...tomorrow I will go to the beach with a friend whose name is XXX. We are going to swim in the sea, I'm going to jump the waves yes... We will eat there. If I can have my toys in the beach... I'm going to make castles in the sand and I'm going to play with the sand."

More info: The girl stuttered a bit, but the content of the story was true, according to the plans of her mother.

<u>Video number 7</u> (duration: 0:38")

"Tomorrow I'm going to a hotel and I will stay that days.... yes Sunday and Monday I think; to sleep... I'm going with XXX, with XXX and with XXX... I want they serve me a burger to dinner, like in the Burger King...

- *Why are you going to a hotel?*

Yes I like it a lot, and maybe they can have a pool"

More info: Apparently, the girl did not understand the question. The content was false (no planned hotel).

<u>Video number 8</u> (duration 0:23")

"Tomorrow I will be in a birthday and I will eat "bocaditos, papas y pelotazos" (Note: a kind of local candy), I will drink coca cola, I will play the hide-and-seek. They are having a "piñata" too (Note: a kind of local playing). It will be in a boat."

More info: The story was true, including the detail of the boat.

<u>Video number 9</u> (duration: 0:41")

"Tomorrow I am going… I'm going to an apartment, with my cousin, which has a pool, we are going to play to throw wa… water balloons, and I want to sleep with my cousin, being together all the time… sleeping together and eating together, doing all with my cousin.

- What will you have for dinner?

I will have a hotdog with a Coca-Cola without caffeine."

More info: The story was false. The girl stuttered a bit.

<u>Video number 10</u> (duration: 0:12")

"Hello my name is XXX. That day he asked me to be his fiancé, and I had to go to the bathroom of the boys for giving him a kiss in the lips."

More info: This was a true story, being contrasted after all with her mother.

<u>Video number 11</u> (duration: 0:30")

"Hello I am XXX. Today my sister is in the cinema with my grandmother, watching Pirates of the Caribbean in 3D. I will go to my grandmother's home to sleep.

- *Do you want to tell us something more?*

No"

More info: A true story according her family

<u>Video number 12</u> (duration: 0:52")

"Every Saturday and Sunday I am going to play tennis in the morning.... Today XXX picked me and after this I went to the playground because every Saturdays at 12:00 there is a show of her instructor. We have... before we did... they have told us a story... then we have painted a drawing, and I've stayed a little bit more to play beside her."

More info: This story was true. The girl stopped from time to time apparently to think about what she was about to say.
